# Neuro-PASC is characterized by enhanced CD4+ and diminished CD8+ T cell responses to SARS-CoV-2 Nucleocapsid protein

**DOI:** 10.3389/fimmu.2023.1155770

**Published:** 2023-05-29

**Authors:** Lavanya Visvabharathy, Barbara A. Hanson, Zachary S. Orban, Patrick H. Lim, Nicole M. Palacio, Millenia Jimenez, Jeffrey R. Clark, Edith L. Graham, Eric M. Liotta, George Tachas, Pablo Penaloza-MacMaster, Igor J. Koralnik

**Affiliations:** ^1^ Ken and Ruth Davee Department of Neurology, Feinberg School of Medicine, Northwestern University, Chicago, IL, United States; ^2^ Department of Microbiology-Immunology, Feinberg School of Medicine, Northwestern University, Chicago, IL, United States; ^3^ Drug Discovery & Patents, Antisense Therapeutics Ltd., Melbourne, VIC, Australia

**Keywords:** COVID-19 immunity, T cell memory, neuro-PASC, IL-6, immunoregulation, proteomics, long COVID

## Abstract

**Introduction:**

Many people with long COVID symptoms suffer from debilitating neurologic post-acute sequelae of SARS-CoV-2 infection (Neuro-PASC). Although symptoms of Neuro-PASC are widely documented, it is still unclear whether PASC symptoms impact virus-specific immune responses. Therefore, we examined T cell and antibody responses to SARS-CoV-2 Nucleocapsid protein to identify activation signatures distinguishing Neuro-PASC patients from healthy COVID convalescents.

**Results:**

We report that Neuro-PASC patients exhibit distinct immunological signatures composed of elevated CD4^+^ T cell responses and diminished CD8^+^ memory T cell activation toward the C-terminal region of SARS-CoV-2 Nucleocapsid protein when examined both functionally and using TCR sequencing. CD8^+^ T cell production of IL-6 correlated with increased plasma IL-6 levels as well as heightened severity of neurologic symptoms, including pain. Elevated plasma immunoregulatory and reduced pro-inflammatory and antiviral response signatures were evident in Neuro-PASC patients compared with COVID convalescent controls without lasting symptoms, correlating with worse neurocognitive dysfunction.

**Discussion:**

We conclude that these data provide new insight into the impact of virus-specific cellular immunity on the pathogenesis of long COVID and pave the way for the rational design of predictive biomarkers and therapeutic interventions.

## Introduction

SARS-CoV-2 is the causative agent of a worldwide pandemic that was first identified in December, 2019. There have been more than 765 million cases and over 6. 9 million deaths worldwide attributable to COVID-19 (1). Although highly effective vaccines are now used to prevent severe acute disease and death caused by SARS-CoV-2, the risk of post-acute symptoms and severe chronic complications after multiple infections is not decreased in vaccinated individuals (2). Therefore, diagnosis and treatment of long-term sequelae after SARS-CoV-2 infection remain an urgent medical concern.

“Long COVID” is defined by the Centers for Disease Control and Prevention (CDC) and others as a wide range of symptoms that can affect the brain, heart, lungs, GI tract, and other body systems lasting more than 4 weeks after disease onset (3). The syndrome has been clinically termed “post-acute sequelae of SARS-CoV-2 infection” (PASC) by the National Institutes of Health and affects an estimated 30% of people infected with SARS-CoV-2 in the U.S (4, 5). “Post-COVID conditions” have also been defined by the World Health Organization as symptoms persisting for more than 3 months that cannot be explained by an alternative diagnosis (6). Neurologic manifestations of PASC (Neuro-PASC) are among the most debilitating and include cognitive dysfunction, fatigue, and many other symptoms leading to decreased quality of life (7–9). They frequently occur in patients with mild initial COVID-19 presentation who never require hospitalization for pneumonia or hypoxemia (10, 11). Despite significant research advances, the underlying causes of Neuro-PASC in these patients remain unclear.

T cell immunity is necessary for host defense against SARS-CoV-2. Severe acute COVID-19 disease was linked to impaired germinal center formation linked to a defective T follicular helper cell response (12), and patients with severe acute disease had higher percentages of immunosuppressive KIR^+^ CD8 T cells (13). Virus-specific T cell responses were also found to be sub-optimal or impaired in severely ill COVID patients (14). Conversely, elevated proportions of proinflammatory T-bet^+^ T cells and memory B cells were associated with lower severity of acute COVID-19 disease (15). Studies in rhesus macaques have additionally shown that CD8^+^ T cell depletion after SARS-CoV-2 infection impairs anamnestic immune protection after subsequent re-infection (16). Though data on immune dysregulation in PASC patients are more limited, recent studies have found autoreactive B cell responses are associated with Neuro-PASC (17), and antiviral effector CD8^+^ T cell responses were significantly diminished in a patient with long-term COVID-19 (18). In addition, patients experiencing persistent post-COVID cognitive impairment had elevated plasma levels of CCL11 and elevated white matter-selective microglial reactivity (19). However, the impact of Neuro-PASC on virus-specific T cell responses remains poorly understood.

Here, we focus on a group of patients who mostly had mild acute disease but subsequently developed Neuro-PASC and a substantial reduction in their quality of life. Our data show four critical findings linking T cell responses with Neuro-PASC symptoms. First, we show that Neuro-PASC patients exhibited enhanced Nucleocapsid-specific T cell responses compared with COVID convalescent controls without persistent symptoms. CD8^+^ memory T cells from Neuro-PASC patients were also less activated and expressed substantially more IL-6 in response to Nucleocapsid protein, which was recapitulated in patient plasma IL-6 levels. Thirdly, the increased severity of cognitive deficits and deterioration of quality-of-life metrics in Neuro-PASC patients were positively correlated with elevated Nucleocapsid-specific T cell responses. Lastly, Neuro-PASC patients presented with elevated immunoregulatory but lower antiviral and T_h_1-inflammatory signatures compared to convalescent controls. Together, these data suggest wide-ranging alterations in anti-Nucleocapsid-specific immune responses in Neuro-PASC patients, with important implications for appropriate diagnostic, prevention, and treatment strategies.

## Methods

### Study design

We aimed to include a robust sample size for every patient group. Data inclusion/exclusion criteria are described below in the *Study participant’s* section. Endpoints were selected prospectively. Replicates for each experiment are described in figure legends.

**Figure 1:**
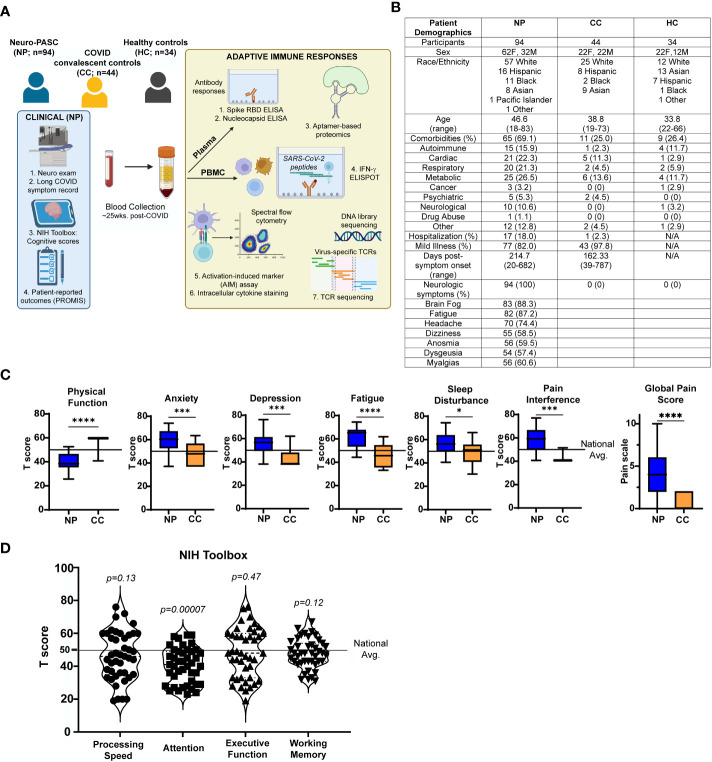
Study design and clinical data **(A)** Study design. **(B)** Demographic table for Neuro-PASC (NP), convalescent controls (CC), and healthy control (HC) study participants. **(C)** PROMIS-57 patient-reported outcome survey T scores for NP patients (n=36) and CC subjects (n=13). **(D)** NIH Toolbox cognitive T scores for NP patients (n = 55). Horizontal black line represents the U.S. national average T score of 50; *p* values relative to demographic-matched US national average by one sample Wilcoxon signed rank test. *p<0.05, ***p<0.005, ****p<0.0001 by two-tailed Student’s t test.

Research objectives were to identify and characterize T cell responses to SARS-CoV-2 linked to Neuro-PASC pathogenesis and specify how these responses differed from COVID convalescent controls without lasting symptoms. We enrolled Neuro-PASC outpatients, convalescent controls, and unexposed healthy controls for our study. Experimental design is outlined in **Figure 1A**. Subjects were not randomized and investigators were not blinded to the study subjects’ grouping prior to conducting experiments and analyzing data.

### Study participants, NIH Toolbox, and PROMIS-57 data collection

We enrolled consenting adult outpatients seen in the Neuro-COVID-19 clinic at Northwestern Memorial Hospital from September 2020-September 2021, including 94 Neuro-PASC patients with documented PCR+ or seropositive IgG results for SARS-CoV-2. In parallel, we recruited 44 healthy COVID convalescents from the surrounding community who tested either PCR+ or seropositive for SARS-CoV-2 before vaccination but had no lingering symptoms lasting >4 weeks; and 34 healthy controls who tested PCR- for SARS-CoV-2 and were also seronegative for IgG against SARS-CoV-2 Spike RBD prior to vaccination. 30 subjects across all 3 groups were vaccinated with the primary series of either the Pfizer BNT162B2 or Moderna mRNA-1273 mRNA vaccines prior to assaying T cells for non-Spike responses. All study subjects remained living throughout the period of observation. Heparinized blood samples were collected one time from each subject at an average of 162.3-214.7 days post-symptom onset (as in **Figure 1B**). Other demographic information, including comorbidity information, is contained in **Figure 1B**. Comorbidities were self-reported and diagnosed prior to SARS-CoV-2 infection. Neuro-PASC patients completed a cognitive function evaluation in the clinic coincident or near the date of their blood sample acquisition with the National Institutes of Health (NIH) Toolbox v2.1 instrument, including assessments of: processing speed (pattern comparison processing speed test); attention and executive memory (inhibitory control and attention test); executive function (dimensional change card sort test); and working memory (list sorting working memory test) (20). PROMIS-57 patient-reported quality of life assessments were administered to Neuro-PASC and COVID convalescent subjects an average of 72 days post-sample collection. Both PROMIS-57 and NIH Toolbox results are expressed as T-scores with a score of 50 representing the normative mean/median of the US reference population and a standard deviation of 10. Toolbox results are adjusted for age, education, gender, and race/ethnicity. Lower cognition T-scores indicate worse performance while higher fatigue, depression, anxiety, or pain interference T-scores indicate greater symptom severity.

### PBMC and plasma collection

30mL of venous blood from study volunteers was collected in blood collection tubes containing sodium heparin from BD Biosciences. Whole blood was layered on top of 15mL of Histopaque 1077 (Sigma-Aldrich) in 50mL Leucosep blood separation tubes (Greiner Bio-One) and spun at 1000g for 18min at RT. Plasma was collected and stored at -80°C. The PBMC layer was collected and washed 2x in sterile PBS before red blood cell lysis with ACK buffer (Quality Biologicals). PBMCs were used in assays either immediately or frozen down for use in the near term, as freezing cells does not significantly affect antigen specific T cell reactivity (21).

### SARS-CoV-2 peptide antigens

**Figure 2:**
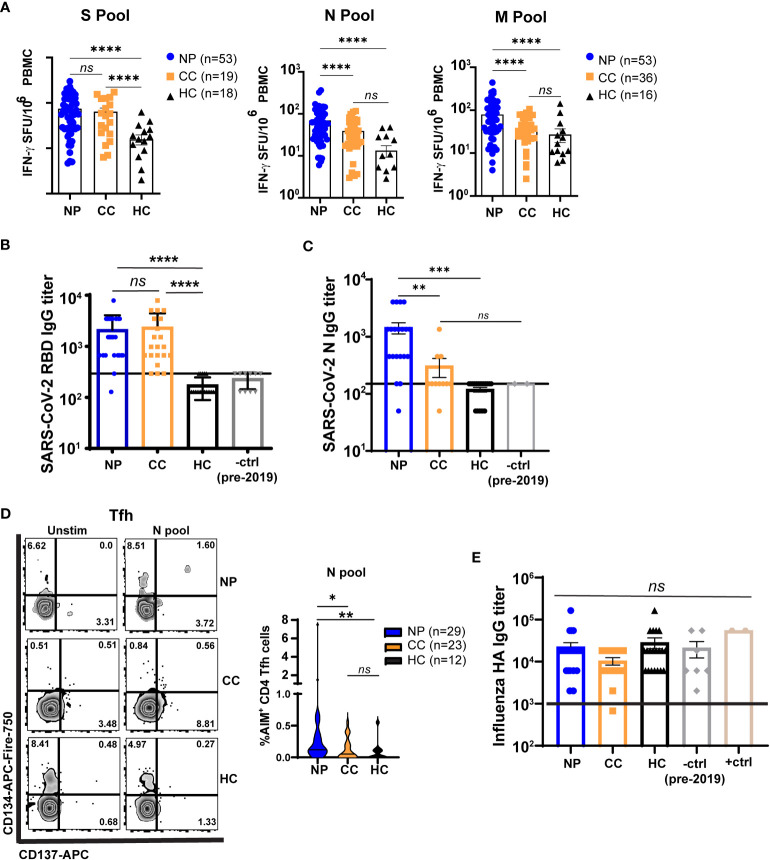
T cells from Neuro-PASC patients have elevated responses to select SARS-CoV-2 structural proteins compared to convalescent controls. **(A)** NP patients and CC subjects display similar IFN-γ responses to SARS-CoV-2 S peptides, but NP patients have enhanced N- and M peptide-specific responses. **(B)** Spike RBD antibody responses are similar in NP and CC subjects. **(C)** NP patients have elevated anti-N IgG titers compared to CC and HC controls. **(D)** NP patients have higher T follicular helper cell (Tfh) activation after N antigen stimulation compared to CC subjects. **(E)** Influenza A Haemagglutinin (HA) antibody responses are similar in all groups. +ctrl = plasma from patients who received the Influenza vaccine within 3 weeks before sample collection; -ctrl = plasma from patients collected pre-2019. Only unvaccinated subjects were examined for anti-Spike responses in A and B Horizontal black line in B,C,E = limit of detection. Data representative of 7 experiments with all conditions plated in duplicate. *p<0.05, **p<0.01, ***p<0.005, ****p<0.0001 by one-way ANOVA with Tukey’s posttest.

All S, N and M peptide arrays used in ELISPOT and flow cytometry studies were obtained from BEI Resources, NIAID, NIH: Peptide Array, SARS-Related Coronavirus 2 Spike (S) Protein; NR-52402, Nucleocapsid (N) Protein, NR-52404; Membrane (M) Protein, NR-52403. The S peptide array consisted of 181 peptides of 13-17aa in length and split into 6 sub-pools (S1-S6) containing 30-31 peptides each. The N peptide array consisted of 59 peptides of 13-17aa each split into 3 sub-pools containing 29-30 peptides each (**Figure 2B**) or with 1 sub-pool further divided into 5 pools of 3-4 peptides each (**Figure 2D**). The M peptide array consisted of 31 peptides of 12-17aa; details in **Figure S1**. All peptides were dissolved in either sterile H_2_O or 50% sterile H_2_O-DMSO up to 1mL for a universal 1mg/mL stock concentration. Peptides were used at a final concentration at 2μg/mL in all assays.

### IgG Spike RBD, nucleocapsid, and haemagglutinin ELISA

Antigen-specific total antibody titers were measured by ELISA as described previously (22). In brief, 96-well flat-bottom MaxiSorp plates (Thermo Scientific) were coated with 1 µg/ml of Spike RBD for 48 hr at 4°C. Plates were washed three times with wash buffer (PBS + 0.05% Tween 20). Blocking was performed with blocking solution (PBS + 0.05% Tween 20 + 2% bovine serum albumin), for 4 hr at room temperature. 6 µl of sera was added to 144 µl of blocking solution in the first column of the plate, 1:3 serial dilutions were performed until row 12 for each sample, and plates were incubated for 60 min at room temperature. Plates were washed three times with wash buffer followed by addition of secondary antibody conjugated to horseradish peroxidase, goat anti-human IgG (H + L) (Jackson ImmunoResearch) diluted in blocking solution (1:1000) and 100 µl/well was added and incubated for 60 min at room temperature. After washing plates three times with wash buffer, 100 µl/well of Sure Blue substrate (SeraCare) was added for 1 min. Reaction was stopped using 100 µl/well of KPL TMB Stop Solution (SeraCare). Absorbance was measured at 450 nm using a Spectramax Plus 384 (Molecular Devices). SARS-CoV-2 RBD and N proteins used for ELISA were produced at the Northwestern Recombinant Protein Production Core by Dr. Sergii Pshenychnyi using plasmids that were produced under HHSN272201400008C and obtained from BEI Resources, NIAID, NIH: Vector pCAGGS containing the SARS-related coronavirus 2, Wuhan-Hu-1 spike glycoprotein gene (soluble, stabilized), NR-52394 and receptor binding domain (RBD), NR-52309, nucleocapsid gene NR-53507. Purified H1 Haemagglutinin protein obtained from BEI Resources, NIAID, NIH: NR-51668.

### Cell stimulation and IFN-γ ELISPOT

Multiscreen-IP plates (Millipore-Sigma) were coated overnight at 4°C with 2μg/mL anti-IFN-γ (clone 1-D1K, Mabtech) washed with sterile PBS, and blocked with complete RPMI-10% FBS. PBMC isolated from Neuro-PASC, COVID convalescent, and healthy control subjects were used either freshly isolated or after thawing and resting overnight in media containing 10ng/μL recombinant human IL-15 (Peprotech) at 37°C, 5% CO_2_. Cells were then plated at a concentration of 2.5x10^5^ cells/well in 200μL of media and stimulated with the indicated antigen mixtures from SARS-CoV-2 at a concentration of 2μg/mL in complete RPMI medium containing 5% human AB serum (Sigma-Aldrich) and 5ng/mL IL-15. Plates were incubated at 37°C, 5% CO_2_ for 20h and washed 5x with dH_2_O and PBS-0.05% Tween-20 (PBS-T). 2μg/mL biotinylated IFN-γ (clone 7-B6-1, Mabtech) diluted in PBS-10% FBS (PBS-F) was added to the respective wells and plates were incubated for 1.5h at RT. Plates were subsequently incubated for 40 minutes at RT in streptavidin-alkaline phosphatase in PBS-F (Jackson ImmunoResearch) was added after washing plates 5x in PBS-T. ELISPOT plates were developed using an Alkaline Phosphatase Conjugate Substrate Kit according to manufacturer’s instructions (Bio-Rad Laboratories, Carlsbad, CA). IFN-γ producing cells were quantified using an ImmunoSpot plate reader (Cellular Technologies, Ltd., Shaker Heights, OH).

### T cell receptor variable beta chain sequencing

**Figure 3:**
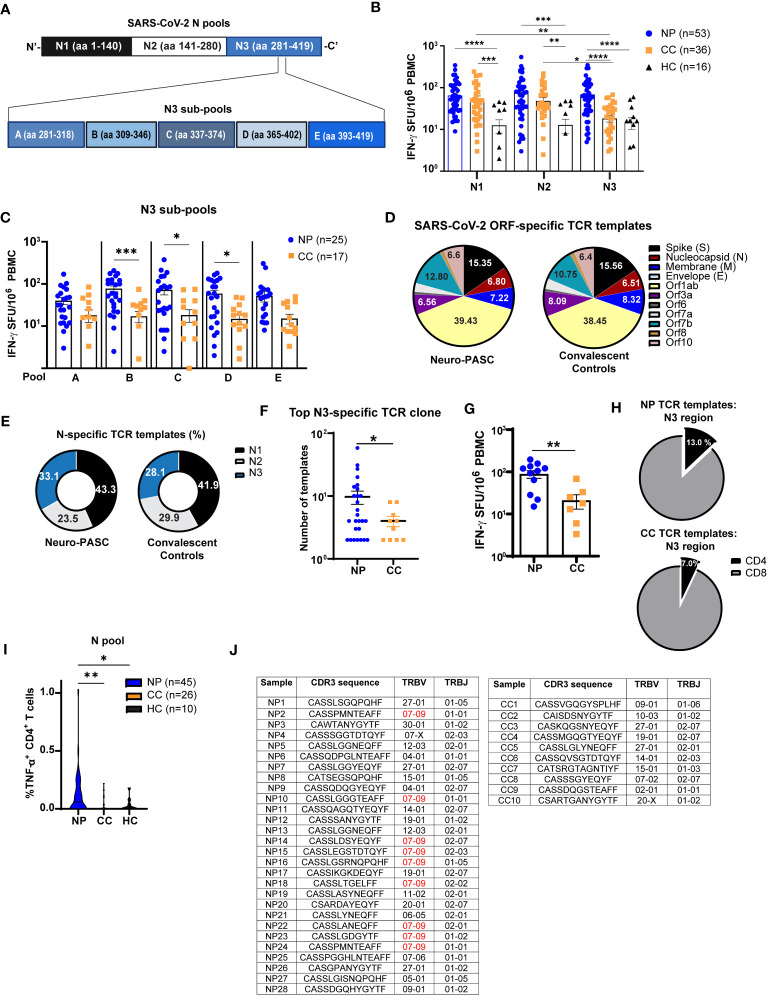
Neuro-PASC patients have elevated reactivity to the C-terminal region of N protein. **(A)** Diagram showing partition of SARS-CoV-2 N peptides into 3 pools comprising the N-terminal (N1), middle (N2), and C-terminal (N3) regions (top) and further splitting of N3 into 5 sub-pools A-E for ELISPOT experiments. **(B)** T cells from NP patients display enhanced reactivity to the C-terminal third of N protein. **(C)** T cell reactivity to N protein is mainly localized to aa 309-402. **(D)** Distribution of SARS-CoV-2-specific TCRs stratified by ORF specificity in unvaccinated NP and CC subjects. **(E)** Elevated proportion of N3-specific TCRs in NP patients. **(F)** Quantification of template copies from the top TCR clone specific for the N3 region in NP and CC subjects. **(G)** N3-specific IFN-γ production from a subset of NP and CC participants in E-F. **(H)** Percentage of N3-specific TCR templates from CD4 vs. CD8 T cells in NP vs. CC. **(I)** N antigen functionally stimulates more TNF-α production from CD4^+^ T cells in NP patients. **(J)** CDR3 sequence, TRBV, and TRBJ usage in top N3 clone from each subject. NP patients have higher TRBV07-09 usage, which is not observed in CC subjects. ELISPOT data combined from 6 independent experiments with the indicated n values. *p<0.05, **p<0.01, ***p<0.005, ****p<0.0001 using one-way ANOVA with Dunnett’s posttest **(B, C, I)** or two-tailed Student’s t Test with Welch’s correction **(F, G)**. ns, not significant.

Immunosequencing of the CDR3, V, and J regions of human TCRβ chains was performed using the immunoSEQ^®^ and T-MAP COVID^®^ Assays (Adaptive Biotechnologies, Seattle, WA). Genomic DNA extracted from individual subjects’ PBMC was amplified in a bias-controlled multiplex PCR, followed by high-throughput sequencing. Sequences were then filtered to identify and quantitate the absolute abundance of each unique TCRβ template for further analysis as previously described (23). TCR specificities to SARS-CoV-2 Spike, Nucleocapsid, Membrane, Envelope, Orf1ab, Orf3a, Orf6, Orf7a, Orf7b, Orf8, and Orf10 were determined using immuneCODE, a publicly available database accessed *via* the immunoSEQ Analyzer platform. Peptide antigens specific for each TCR from immuneCODE were then aligned to the Nucleocapsid amino acid sequence to demarcate regional specificity (“N1” vs. “N2” vs. “N3”). The value for the top expanded N3-specific TCR clone was counted for each NP and CC subject for **Figure 3E**. HLA typing was done by the bioinformatics group at Adaptive Biotechnologies through their HLA classifier platform (**Figures S4, S5**).

### Antibodies and flow cytometry

Fresh or frozen PBMCs isolated from the indicated patient groups were stimulated with antigen mixtures as above for 20-22h at 37°C, 5% CO_2._ For intracellular staining and cytokine detection, the Brefeldin-A Golgi plug (Biolegend) was added at a 1:1000 concentration 2 hours after antigenic stimulation commenced. Cells were washed with PBS-1% BSA after incubation and stained with the indicated antibodies for surface phenotyping by AIM assay or for intracellular cytokine staining (ICS; antibodies used described in **Supplemental Table S1**). Cells from each subject were left unstimulated in medium containing 5ng/mL IL-15 (“background”) or stimulated in the presence of the indicated antigens. Fixation and permeabilization was performed using Cytofix/Cytoperm (BD Biosciences). Surface staining was done in the dark at 4°C for 30 minutes, while ICS was done in the dark at RT for 45 minutes. Flow cytometry was conducted on 2-5x10^5^ cells per condition. Data was acquired on a BD FACSymphony Spectral analyzer and analyzed using FlowJo v10 (BD Biosciences) and SPICE-Pestle (24).

### SomaScan profiling

**Figure 4:**
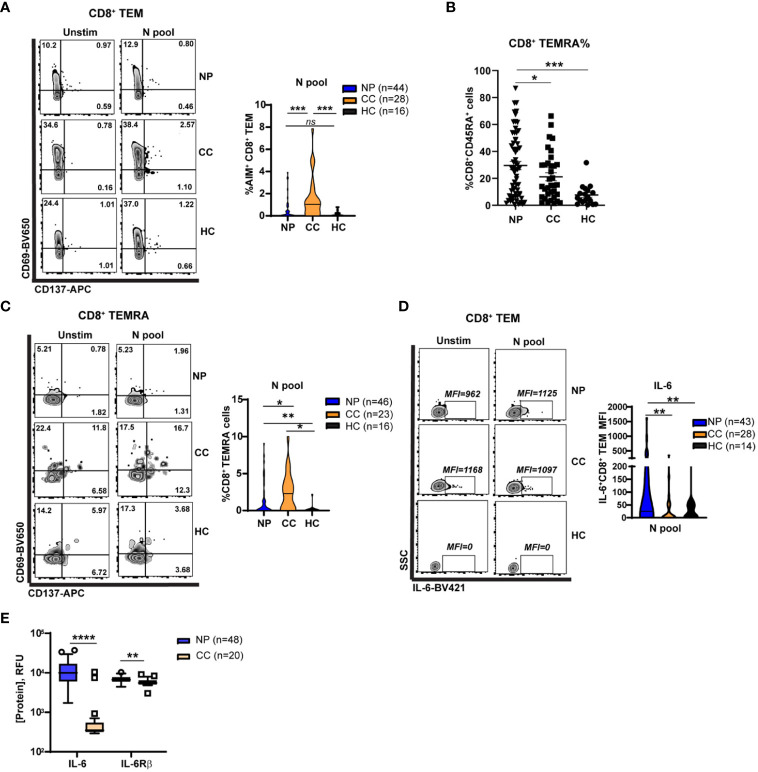
Elevated Nucleocapsid-specific CD4^+^ T cell and attenuated CD8^+^ memory T cell activation in Neuro-PASC patients. **(A)** CD4^+^ T cells from NP patients have enhanced N antigen-specific TNF-α production compared to CC. **(B)** CD8^+^ TEM from NP patients have enhanced IL-6 production after N antigen stimulation compared to CC subjects on a per-cell basis (mean fluorescence intensity; MFI). **(C)** Increased soluble IL-6 and IL6Rβ in NP patient plasma compared with CC subjects. **(D)** CD8^+^ TEM from NP patients show decreased activation after stimulation with N peptides. **(E)** Higher percentages of CD8^+^ TEMRA cells are found in NP patients compared to control groups. **(F)** CD8^+^ TEMRA cells from NP patients are less activated by N antigens compared with CC subjects. Data combined from 5 independent experiments with the indicated n values. *p<0.05, **p<0.01, ***p<0.005, ****p<0.0001 using two-tailed Student’s t test with Welch’s correction. ns, not significant.

Heparinized plasma from 48 Neuro-PASC patients and 20 healthy COVID convalescents whose T cell and antibody responses were characterized in **Figures 2**–**4** were assayed for the presence of more than 7,000 proteins using the SOMAscan proteomics platform. The SOMAscan assay is a sensitive, high-throughput technique that uses chemically modified DNA aptamers to specifically bind and quantify proteins of interest from very small quantities of plasma (25). The assay measures a wide range of receptors, intracellular signaling proteins, growth factors, and secreted proteins. All plasma samples were analyzed at SomaLogic Operating Co, Inc. (Boulder, CO).

**Figure 5:**
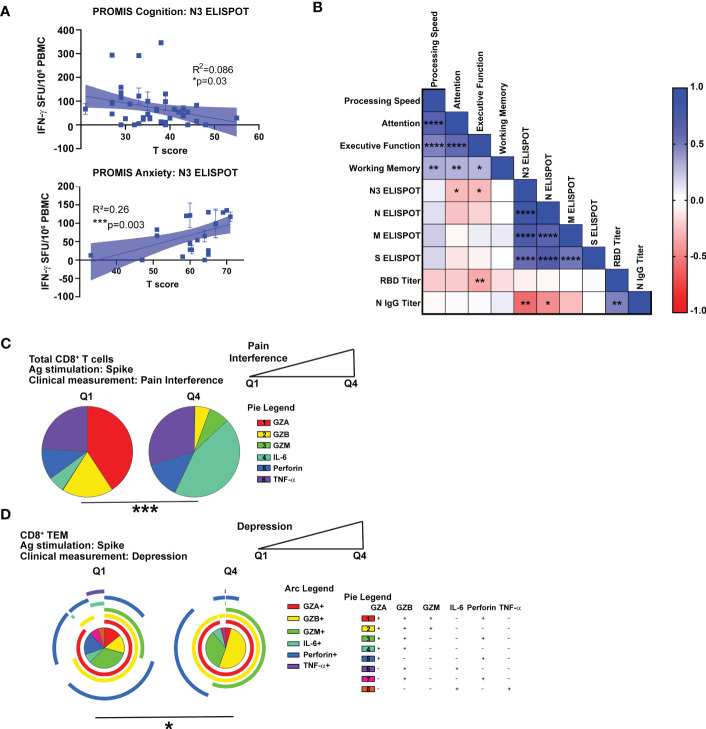
Correlation of cognitive and psychiatric clinical measures with virus-specific immune responses in Neuro-PASC patients. **(A)** N3-specific IFN-γ production is negatively correlated with self-reported cognition scores (top) and positively correlated with anxiety scores (bottom) in NP patients. **(B)** NP patients with lower scores on Attention or Executive Function cognitive tests had higher N3-specific IFN-γ responses and RBD IgG titers. **(C)** High Pain Interference scores correlate with more IL-6 production from CD8^+^ T cells in response to S peptides. **(D)** High depression scores correlate with lower polyfunctionality in CD8^+^ TEM. Data representative of 5 independent experiments with n=39-51 for correlation data analysis **(A, B)** and n=8-9 NP subjects per quartile for SPICE analysis **(C, D)**. Correlations calculated using simple linear regression **(A)**, nonparametric Spearman rank correlation **(B)**, or Permutation test **(C, D)**. All pie graphs are background subtracted (unstimulated conditions). *p<0.05, **p<0.01, ***p<0.005, ****p<0.001.

For statistical comparison, all relative fluorescence unit (RFU) values for individual proteins were first analyzed by Gene Set Enrichment Analysis (GSEA version 4.2.3; Broad Institute; Molecular Signatures Database: hallmark, curated, KEGG, and reactome gene sets) to determine significantly enriched pathways between NP and CC groups (**Figure 5A**). The false discovery rate cutoff was 0.05. RFUs for proteins belonging to a particular pathway (immunoregulatory or TASOR antiviral) that were enriched in NP or CC were then analyzed using two-tailed t-Test (**Figure 5B**). Within-group correlations for Neuro-PASC symptoms with individual protein concentrations were determined using Pearson correlation (**Figure 5C**).

### Quantification and statistical analysis

**Figure 6:**
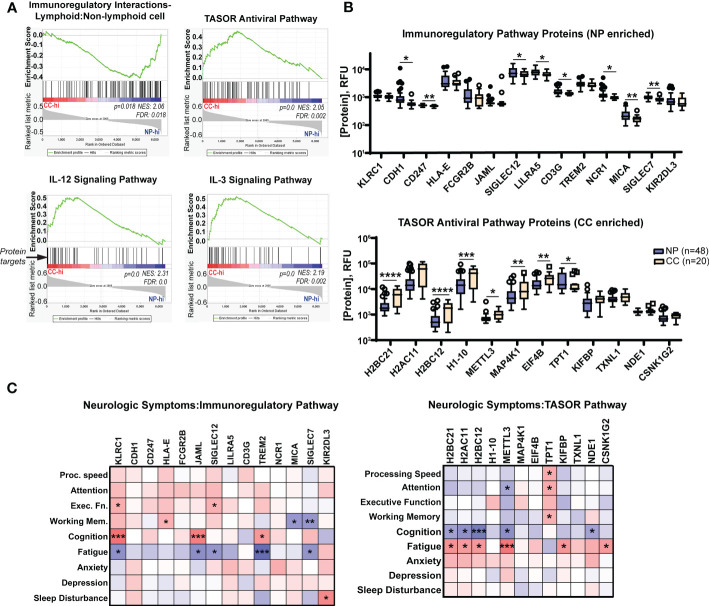
Neuro-PASC patients have elevated immunoregulatory and decreased antiviral response-associated proteins in plasma correlating with enhanced symptom severity and cognitive dysfunction. **(A)** Gene set enrichment analysis (GSEA) demonstrating elevations in immunoregulatory pathway-related proteins (top left panel) in NP patients contrasting with elevated pro-inflammatory and antiviral pathway-related proteins (top right, bottom panels) in CC subjects. List of proteins analyzed in each pathway found in **Tables S2**–**S5**. **(B)** Quantification of individual immunoregulatory (top) and TASOR antiviral pathway-associated protein levels (bottom) between Neuro-PASC patients and healthy COVID convalescents. **(C)** Patient-reported outcomes of symptom severity and cognitive scores are significantly correlated with expression levels of immunoregulatory proteins (left) and TASOR pathway proteins (right). RFU: relative fluorescence units. FDR: false discovery rate. NES: normalized enrichment score. *p<0.05; **p<0.01; ***p<0.005; ****p<0.0001 by Student’s t test **(B)** or Pearson correlation **(C)**.

Statistical tests to determine significance are described in figure legends and conducted largely in Prism (GraphPad). For pie graphs in **Figure 6**, SPICE analysis was used to determine statistical significance. SPICE is a data-mining software application that analyzes large FLOWJO datasets from polychromatic flow cytometry and organizes the normalized data graphically. SPICE defines a statistic for the nonparametric comparison of complex distributions based on multi-component measurements (24). For pie graphs or heatmaps generated using SPICE software analysis, statistics were determined by Permutation test following unstimulated background subtraction, with additional thresholding of 0.03% to account for noise, using SPICE-Pestle. *p*-values lower than 0.05 were considered statistically significant. Quartile stratification was performed within group for the Neuro-PASC cohort in **Figures 6C–**F. Clinical data were collected and managed using REDCap electronic data capture tools hosted at Northwestern University Feinberg School of Medicine. All error bars on figures represent values ± SEM.

### Study approval

This study was approved by the Northwestern University Institutional Review Board (Koralnik Lab, IRB STU00212583). Informed consent was obtained from all enrolled participants. Samples were de-identified before banking.

## Results

### Clinical characteristics of Neuro-PASC patients and control participants

We enrolled a total of 172 participants, including 144 prior to SARS-CoV-2 vaccination and 28 participants post-vaccination. We recruited from the Neuro-COVID-19 outpatient clinic at Northwestern Memorial Hospital or from the surrounding Chicago area. Patients were enrolled on a rolling basis as they were seen in the clinic. These included 94 Neuro-PASC patients (“NP”; confirmed RT-PCR+ or anti-SARS-CoV-2 Spike IgG+) meeting Infectious Disease Society of America clinical criteria for COVID-19 from March, 2020 until October, 2021 who had neurologic symptoms lasting at least 6 weeks post-infection, as previously reported (9). Among those, 77 (82.0%) were never hospitalized for pneumonia or hypoxia and had mild disease. We additionally recruited 44 healthy COVID convalescents without symptoms persisting more than 4 weeks from onset, including 43 (97.8%) who had mild acute presentation not requiring hospitalization, hereby referred to as “convalescent controls (CC; RT-PCR+ or seropositive for anti- SARS-CoV-2 Spike IgG pre-vaccination). We also included 34 healthy controls who were unexposed to SARS-CoV-2 (“HC”; RT-PCR- and seronegative for SARS-CoV-2 Spike-IgG pre-vaccination. Study design is shown in **Figure 1A**.

Neuro-PASC patients displayed a constellation of neurological symptoms similar to those previously reported (26) such as headache, fatigue, brain fog, and myalgia (**Figure 1B**). Neuro-PASC patients scored significantly lower on physical function and higher on anxiety, depression, fatigue, sleep disturbance, and pain interference measures compared with convalescent controls or the U.S. national average on patient reported outcomes (PROMIS-57) (27) surveys (**Figure 1C**). NIH toolbox tests objectively assessing cognitive function (20) also found Neuro-PASC patients to have significantly lower scores in the attention domain, indicating cognitive dysfunction relative to a demographic-matched U.S. population (**Figure 1D**).

### Alterations in T cell responses to SARS-CoV-2 nucleocapsid protein in Neuro-PASC

T cell responses to SARS-CoV-2 structural proteins were determined by IFN-γ ELISPOT. Peripheral blood mononuclear cells (PBMC) from each subject were stimulated with overlapping peptides from the Spike (S), Nucleocapsid (N), or Membrane (M) structural proteins of SARS-CoV-2 (**Figure S1**). We first determined whether the severity of acute disease affected virus-specific T cell responses in our Neuro-PASC cohort. Importantly, hospitalization during acute SARS-CoV-2 infection did not impact cognitive scores (**Figure S2A**) or T cell responses (**Figure S2B**), justifying the inclusion of post-hospitalized Neuro-PASC patients in our subsequent analyses. IFN-γ^+^ responses to S peptides were similar between Neuro-PASC patients and convalescent controls (**Figure 2A** left panel). However, Neuro-PASC patients exhibited higher IFN-γ^+^ responses against N and M peptides (**Figure 2A**, middle and right panels) compared with convalescent controls. Though antibody responses to Spike receptor-binding domain (RBD) did not differ between groups (**Figure 2B**), Neuro-PASC patients had significantly higher antibody responses to N protein (**Figure 2C**) and higher N-specific T follicular helper cell (Tfh) responses that facilitate antibody class-switching (**Figure 2D**). This was determined by the activation-induced marker assay (AIM; CD134^+^CD137^+^ Tfh cells; gating in **Figure S7C**) which measures cytokine-independent, antigen-specific, TCR-mediated T cell activation and has been previously used to detect SARS-CoV-2-specific CD4^+^ (CD137^+^CD134^+^) and CD8^+^ (CD69^+^CD137^+^) T cells (28). No differences in antibody titers were found against the irrelevant Haemagglutinin protein from Influenza virus (**Figure 2D**), demonstrating immune responses were SARS-CoV-2-specific. These data show that N-specific T cell and antibody responses are elevated in those with Neuro-PASC compared to convalescent controls.

### Enhanced IFN-γ production and CD4^+^ T cell activation to C-terminal region of N protein in Neuro-PASC

Having shown elevated IFN-γ responses to N protein in Neuro-PASC patients, we queried whether specific regions of N protein enhanced T cell activation activation. We focused on N over M protein because N-specific immune responses can persist for up to 12 months post-infection (29), and Neuro-PASC patients were enrolled at an average of 7 months post-infection allowing for accurate detection. As the early roll-out of COVID-19 vaccines in our area made it difficult to find unvaccinated individuals after January 2021, we used both vaccinated and unvaccinated subjects in these experiments to increase our sample size. There were no differences in T cell or antibody responses to N protein before and after vaccination with SARS-CoV-2 Spike protein (**Figure S3**). Nucleocapsid peptides were divided into 3 antigen pools (**Figure 3A**, top), and elevated IFN-γ responses in Neuro-PASC patients were traced to the C-terminal region of the protein (N3; **Figure 3B**), particularly within amino acids 309-402 (**Figures 3A–C**).

To confirm these findings, we performed T cell receptor sequencing (TCR-Seq) on a subset of unvaccinated Neuro-PASC patients and convalescent controls using the ImmunoSEQ™ platform from Adaptive Biotechnologies. Employing the COVID classifier tool in the ImmunoSEQ™ Analyzer, we identified total numbers of TCR templates specific for individual ORFs from SARS-CoV-2. No significant differences in the relative percentages of ORF-specific TCRs were found between groups (**Figure 3D**). However, when N peptide antigens were assigned to the regions shown in **Figure 3A**, a greater percentage of TCR reactivity mapped to the N3 region (**Figure 3E**) due to enhanced N3-specific TCR expansion in Neuro-PASC patients (**Figure 3F**). IFN-γ ELISPOT data from study participants included in the TCR-Seq analysis corroborated these findings (**Figure 3G**). Elevated N-specific T cell responses in Neuro-PASC patients were primarily due to expanded CD4^+^ T cell reactivity (**Figures 3H, I**). Interestingly, 9 of 28 (32.1%) of NP patients used TRBV07-09 in the top N3-specific TCR clone (**Figure 3J**). In contrast, none of the 10 CC participants used the same TCR β gene. No significant HLA-A, -B, -DP, -DQ- or -DR skewing was observed in either group (**Figures S4, 5**), though Neuro-PASC patients had more HLA diversity potentially due to their higher numbers in the analysis. Taken together, these data show enhanced TCR clonal expansion to the C-terminal region of the N protein in Neuro-PASC patients that could not be explained by differences of HLA alleles between the two groups.

### Attenuated N-specific CD8^+^ memory T cell activation in Neuro-PASC patients

CD8^+^ memory T cells are important for effective anti-viral immunity and can persist for several years after the related SARS-CoV-1 infection (30). However, little is known about whether CD8^+^ memory T cell function is altered in Neuro-PASC. Immunophenotyping showed no differences between groups in total percentages of most T cell subsets in the unstimulated condition (**Figure S6**); therefore, we conducted functional assays investigating T cell memory responses. CD8^+^ T effector memory cells (TEM or TEMRA; gating strategy in **Figure S7A**), poised for rapid cytotoxic function upon antigen re-encounter, exhibited significant N-specific activation in convalescent controls but not in Neuro-PASC patients (**Figure 4A**). Percentages of CD8^+^ TEMRA cells were significantly elevated in Neuro-PASC patients (**Figure 4B**) but less activated by N antigens compared with convalescent controls (**Figure 4C**). N peptides also promoted higher IL-6 production in CD8^+^ TEM from Neuro-PASC patients compared to convalescent controls (**Figure 4D**; FMO in **Figure S7B**). Similarly, Neuro-PASC patients had significantly higher plasma levels of IL-6 and IL-6 receptor β (IL-6Rβ) when evaluating serum samples from patients in **Figure 4D** (**Figure 4E**). Stimulation of PBMCs with a pool of overlapping peptides comprising S, N, and M proteins likewise showed enhanced IL-6 production from monocytes and neutrophils from Neuro-PASC patients compared with convalescent controls (**Figure S8**). These results suggest that Neuro-PASC patients have decreased N-specific CD8^+^ recall responses but enhanced IL-6 production to N antigens compared to convalescent controls.

### Impaired cognition and decreased quality of life metrics correlate with distinct patterns of virus-specific T cell activation

We next determined if within-group differences in antiviral immune responses correlated with clinical measures of symptom severity in Neuro-PASC. Lower cognitive scores and higher anxiety scores were correlated with high levels of IFN-γ-stimulated by N3 peptides (**Figure 5A**). Correlation analyses further demonstrated negative correlations between attention and executive function scores and IFN-γ responses to the N3 region as well as RBD-specific antibody responses (**Figure 5B**). To determine associations between clinical scores and T cell effector functions, we partitioned T scores from NIH Toolbox or PROMIS-57 measurements (**Figures 1C, D**) into quartiles and used only the lowest and highest groups (Q1 vs. Q4) for analysis. Neuro-PASC subjects reporting high degrees of pain produced significantly more IL-6 and less cytotoxic effector molecules from CD8^+^ T cells than those with low pain scores (**Figure 5C**). Further, patients reporting high depression scores had elevated virus-specific granzyme production (**Figure 5D**). Taken together, these data show correlations between cognitive dysfunction and impaired quality of life and altered patterns of CD8^+^ T cell effector functions.

### Enrichment in immunoregulatory proteins and reduction in antiviral- response proteins in Neuro-PASC patients correlate with cognitive dysfunction

The multiplexed proteomics platform SOMAscan has been successfully used in previous studies to identify biomarkers associated with conditions such as hepatocellular carcinoma (31), Alzheimer’s disease (32), and drug treatment of myocardial infarction (33). The technology utilizes the natural 3D folding of single-stranded DNA-based protein recognition aptamers to quantify levels of more than 7000 unique proteins in biological fluids (25). We used this platform to determine whether Neuro-PASC patients had distinct plasma proteomic signatures through pathway analysis as well as comparison of individual protein levels. Gene set enrichment pathway analysis (GSEA) has previously been used on proteomics data to identify dysregulated circuits in complex disease states (34). GSEA similarly identified an enrichment in immunoregulatory pathway proteins in Neuro-PASC patients and conversely, elevated antiviral and T_h_1-type inflammatory pathway proteins such as IL-12 and IL-3 in convalescent controls (**Figure 6A**). Comparison of individual proteins enriched in the immunoregulatory pathway identified significantly elevated CDH1 (E-cadherin), SIGLEC7, MICA, and other molecules involved in inhibiting T cell function (35) (**Figure 6B**, top panel). In contrast, plasma from convalescent controls were enriched in the antiviral TASOR pathway proteins H2BC12, METTL3, and MAP4K1 (**Figure 6B**, bottom panel), among others, which are involved in preventing intracellular viral replication and T cell differentiation (36). A number of protein targets were correlated with cognitive performance or neurologic symptom severity in both pathways (**Figure 6C**), with a particularly significant negative correlation between self-reported cognition scores and expression of the inhibitory NK cell/CD8^+^ T cell receptor KLRC1 (**Figure 6C**, left panel) and conversely, positive outcome correlations for those expressing high levels of METTL3, an important driver of T cell differentiation from the naïve state. These results highlight the interconnection of enhanced immunoregulatory and reduced antiviral pathway signatures with cognitive dysfunction in Neuro-PASC patients.

Overall, our study demonstrates that Neuro-PASC patients have elevated T cell responses to the C-terminal region of Nucleocapsid protein, impaired N-specific CD8^+^ memory responses, and elevated N-specific IL-6 production compared with convalescent controls. In addition, we show unique correlations between cognitive dysfunction and quality of life impairments and increased N-specific T cell responses, suggesting that elevated virus-specific T cell responses are not always linked to better clinical outcomes if directed against N protein. Importantly, proteomics analysis found upregulations in immunoregulatory signatures and downregulation in inflammatory and antiviral response signatures in Neuro-PASC patients that were highly correlated with neurocognitive dysfunction. Altogether, we show that Neuro-PASC patients exhibit distinct SARS-CoV-2-specific T cell responses that may facilitate identification and treatment of long COVID.

## Discussion

We identified a distinct pattern of T cell activation in Neuro-PASC patients which provides novel insights into Neuro-PASC pathogenesis using multimodal analyses including TCR sequencing and evaluation of the plasma proteome. Prior studies have either focused on characterizing T cell responses to acute infection in COVID convalescents broadly as opposed to those with PASC (37, 38), or on immunophenotyping and autoantibody responses in Neuro-PASC patients (17). We aimed to fill this knowledge gap and examine how virus-specific T cell responses in patients with Neuro-PASC may differ from healthy convalescents and contribute to neurologic symptom severity.

### Proposed mechanisms for Neuro-PASC

Several hypotheses have been put forward defining the underlying mechanisms of Neuro-PASC. One theory is that Neuro-PASC symptoms may be caused by direct infection of the CNS, though studies have been equivocal. SARS-CoV-2 may gain entry into the CNS through the olfactory bulb, a theory supported by the presence of viral protein in neurons from post-mortem autopsies and live virus in the brain in mouse models (39, 40). However, other studies were unable to find evidence of SARS-CoV-2 in the CNS of patients who died with neurologic symptoms (41) or in the cerebrospinal fluid (CSF) (42), suggesting that infection of the nervous system may be transient or may not occur in all infected individuals. Importantly, SARS-CoV-2 RNA or intrathecally-produced antiviral antibodies were undetectable in the CSF at 90 days post-infection in Neuro-PASC patients (43) which suggests that direct CNS infection may not be the underlying cause of Neuro-PASC. Despite this, autopsy studies have identified persistent viral RNA or antigen in extra-respiratory sites other than the brain (44), and some patients have been found to test N-antigen positive in the nasopharynx for months after acute infection while experiencing long COVID symptoms (45). We also found that Neuro-PASC patients had elevated anti-N antibody titers though we obtained their samples more than 7 months after acute infection when anti-N antibody titers would fall below detection in most COVID convalescents (46). This is suggestive of N antigen or viral persistence in cryptic reservoirs, but future studies are needed to evaluate the presence of infectious virus, preferably using highly sensitive quantification techniques such as viral outgrowth assays (47). However, clinical trials to test SARS-CoV-2 antiviral drugs in the treatment of long COVID have already begun (48), demonstrating traction for the persistent infection hypothesis within the medical and research communities.

### Enhanced T cell reactivity to the C-terminal region of SARS-CoV-2 N protein

Neuro-PASC patients displayed high IFN-γ responses to the C-terminal domain of N protein (N3 region) while convalescent controls had limited reactivity. It is possible that N3-specific T cell responses remain high Neuro-PASC patients due to increased CD4^+^ T cell clonal expansion and cytokine production compared with convalescent controls, which we found in our cohort. Though increased antiviral T cell responses may ordinarily be thought to be protective, studies have found conflicting associations between increased SARS-CoV-2 T cell responses and COVID-19 disease outcomes. Virus-specific TCR expansion was higher in COVID convalescents with more severe acute disease (49). However, another group found that elevated T cell responses to an N-terminal peptide from N protein in patients expressing HLA-B*07-02 had less severe acute disease (50). However, neither study determined whether convalescent subjects had PASC at the time of sample collection, and we found no significant differences in the prevalence of HLA-B*07-02 between groups. Further research is needed to determine whether enhanced T cell responses to the C-terminal region of N protein in Neuro-PASC patients are detrimental to patient outcomes, which may inform vaccination and treatment strategies.

### Attenuated CD8^+^ T cell memory responses and increased IL-6 in Neuro-PASC patients

Effective generation of T cell memory responses can be important to protect against future infections with the same pathogen. CD8^+^ T effector memory (TEM) cells from Neuro-PASC patients displayed reduced antigen-specific activation compared with convalescent controls, suggestive of a diminished effector response. The costimulatory molecule CD137 may play a role in this because it provides necessary orthogonal signal activating virus-specific T cells (51), but this marker was reduced on CD8^+^ memory T cells from Neuro-PASC. Prior studies have shown that asymptomatic individuals display a robust T cell recall response to SARS-CoV-2 Nucleocapsid protein after infection (38), suggesting that the lack of T cell memory responses in Neuro-PASC patients is detrimental. We also observed a significant elevation in CD8^+^ TEMRA cells in Neuro-PASC patients compared to control groups. CD8^+^ TEMRA cells can accumulate during persistent viral infections and contribute to immunosenescence (52). Their decreased virus-specific activation in Neuro-PASC patients suggests lower cytotoxic capacity compared with convalescent controls. Our data suggest that CD8^+^ TEMRA cells may be functionally anergic in Neuro-PASC patients compared with convalescent controls and may contribute to the pathogenesis of PASC.

Significantly, CD8^+^ TEM from Neuro-PASC patients expressed higher levels of IL-6 in response N antigens which was recapitulated in unstimulated patient plasma compared to convalescent controls. CD8^+^ T cell expression of IL-6 was also significantly correlated patient-reported pain scores. IL-6 can play a regulatory role in T cell responses during viral infections by suppressing T_h_1 differentiation (53), and promoting pathogen survival while exacerbating clinical disease in SARS-CoV-1 infection (54). In fact, blocking IL-6 activity enhances virus-specific CD8^+^ T cell immunity (55), and overexpression of IL-6 can lead to viral persistence by impairing CD8^+^ lytic functions (56) and the development of CD8^+^ T cell memory (57). Indeed, high serum levels of IL-6 were associated with poor clinical outcomes in severely ill COVID-19 patients (37). IL-6 has also been associated with fatigue and cognitive impairment in a cohort of PASC patients who had mild acute infection (58). Thus, our data suggest that enhanced IL-6 production by CD8^+^ T cells may be involved in the etiology or pathogenesis of Neuro-PASC and open new avenues of research for the treatment of long COVID by blocking IL-6 activity.

### Antiviral immune responses correlate with impaired cognition and lower quality of life in Neuro-PASC

Neuro-PASC patients reported significantly elevated levels of anxiety, depression, pain, and other symptoms compared with convalescent controls. The severity of these deficits was correlated with antiviral adaptive immune responses, and it is possible that T cells can contribute to these symptoms. Studies in rodents have shown that T cell responses can affect the severity of pain and analgesia (59); it may follow that particular T cell activation patterns can be linked to high pain scores in Neuro-PASC. Inflammation-related transcriptional programs are also differentially regulated in T cells from patients with depression (60), providing a possible link between enhanced granzyme production and elevated depression scores. Thus, the association of SARS-CoV-2-specific cytokine signatures with the severity of Neuro-PASC symptoms may provide predictive value in terms of clinical outcomes.

### Elevated immunoregulatory and lower antiviral signatures in Neuro-PASC patients

Proteomic analysis demonstrated that Neuro-PASC patients had relatively blunted inflammatory and antiviral response signatures compared to convalescent controls, while simultaneously having elevated immunoregulatory protein expression. Further analyses at the individual protein level showed upregulation of immunoregulatory proteins such as NCR1 involved in T cell suppression of antiviral CD8^+^ T cell responses (61). These data support our findings showing decreased antiviral CD8^+^ T cell recall responses and suggest that an imbalance between immunoregulatory and antiviral pathways may play a role in Neuro-PASC pathogenesis. In line with this, one of the strongest associations we found with poor cognitive scores involved the NK and CD8^+^ T cell inhibitory receptor KLRC1 that downregulates cytotoxic capacity (62). KLRC1 expression on CD8^+^ T cells is upregulated by IL-6 (63), and enhanced KLRC1 expression has been found on exhausted CD8^+^ T cells from acute COVID-19 patients (64). Based on our data, it is therefore possible that enhanced IL-6 production from CD8^+^ T cells may upregulate KLRC1 and suppress CD8^+^ T cell function in Neuro-PASC patients, which may increase Neuro-PASC symptom severity. Together, these data illuminate a specific T cell signature composed of decreased CD8^+^ T cell memory responses and increased IL-6 stimulated by Nucleocapsid protein antigens that associate with Neuro-PASC.

## Limitations of study

One limitation is the relatively small sample size of unvaccinated convalescent control subjects. This was due to the wide implementation of SARS-CoV-2 vaccines in Chicago area soon after beginning study enrollment. Another limitation was not being able to control for time of sample collection with respect to date of COVID-19 symptom onset because we recruited patients on a rolling basis as they were seen in the Neuro-COVID clinic. Additionally, as we hypothesize that Neuro-PASC could be the result of a persistent or protracted infection, future studies would require testing of potential cryptic viral reservoirs, including stool or post-mortem multi-organ tissue sampling from Neuro-PASC patients.

## Data availability statement

The full datasets generated in the current study are available from the corresponding author upon request. TCR sequencing data can be found in an online repository: GEO, accession number GSE225942.

## Author contributions

Conceptualization LV and IK. Investigation LV, BH, ZO, PL, NP and GT. Formal Analysis LV, BH, MJ, EL, P-PM and NP. Resources LV, GT, P-PM, IK, Data curation LV, EG, JC. Writing LV with feedback from all authors. Supervision LV, P-PM and IK. Project administration LV. Funding acquisition LV, P-PM, and IK. All authors contributed to the article and approved the submitted version.

## Supplementary material

Supplementary Table 1 Antibodies used in study.

Supplementary Table 2 Proteins associated with the enriched Reactome term “Immunoregulatory interactions between a lymphoid and non-lymphoid cell”.

Supplementary Table 3 Proteins associated with the enriched term “TASOR target genes”.

Supplementary Table 4 Proteins associated with the enriched Reactome term “Interleukin 12 family signaling”.

Supplementary Table 5 Proteins associated with the enriched WP term “IL3 signaling pathway”.
