# Corrigendum: Neuro-PASC is characterized by enhanced CD4+ and diminished CD8+ T cell responses to SARSCoV-2 Nucleocapsid protein

**DOI:** 10.3389/fimmu.2023.1275925

**Published:** 2023-08-24

**Authors:** Lavanya Visvabharathy, Barbara A. Hanson, Zachary S. Orban, Patrick H. Lim, Nicole M. Palacio, Millenia Jimenez, Jeffrey R. Clark, Edith L. Graham, Eric M. Liotta, George Tachas, Pablo Penaloza-MacMaster, Igor J. Koralnik

**Affiliations:** ^1^Ken and Ruth Davee Department of Neurology, Feinberg School of Medicine, Northwestern University, Chicago, IL, United States; ^2^Department of Microbiology-Immunology, Feinberg School of Medicine, Northwestern University, Chicago, IL, United States; ^3^Drug Discovery & Patents, Antisense Therapeutics Ltd., Melbourne, VIC, Australia

**Keywords:** COVID-19 immunity, T cell memory, neuro-PASC, IL-6, immunoregulation, proteomics, long COVID

In the published article, there was an error in **Figure 4** as published. The figure legend did not match the figure panels. The corrected **Figure 4** and its caption appear below.

**Figure 4:**
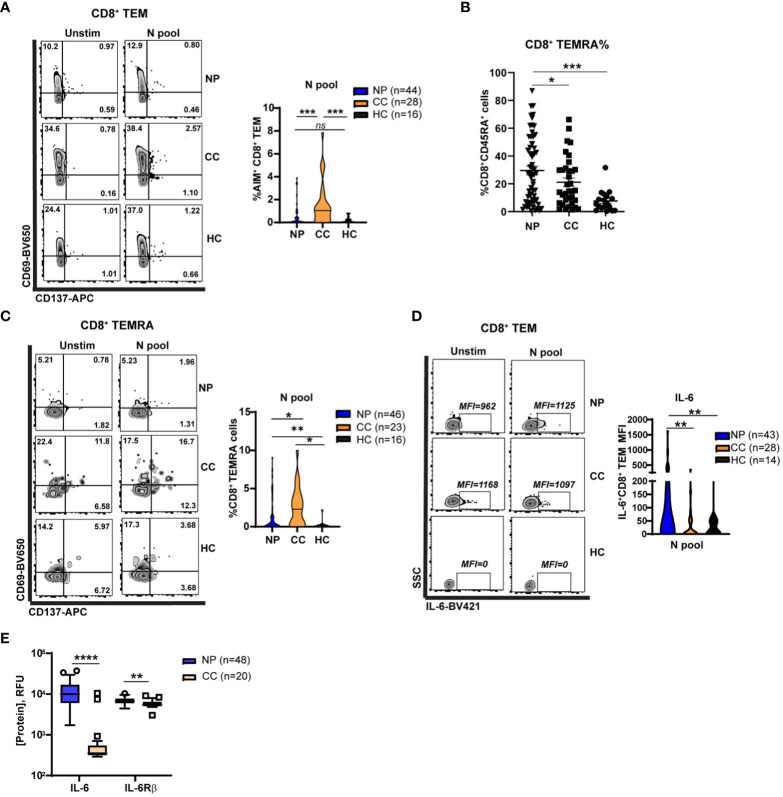
Elevated Nucleocapsid-specific CD4^+^ T cell and attenuated CD8^+^ memory T cell activation in Neuro-PASC patients **(A)** CD8+ TEM from NP patients show decreased activation after stimulation with N peptides. **(B)** Higher percentages of CD8^+^ TEMRA cells are found in NP patients compared to control groups. **(C)** CD8+ TEMRA cells from NP patients are less activated by N peptides compared with CC subjects. **(D)** CD8^+^ TEM from NP patients have enhanced IL-6 production after N antigen stimulation compared to CC subjects on a per-cell basis (mean fluorescence intensity; MFI). **(E)** Increased soluble IL-6 and IL6Rβ in NP patient plasma compared with CC subjects. Data combined from 5 independent experiments with the indicated n values. *p<0.05, **p<0.01, ****p<0.0001 using two-tailed Student’s t test with Welch’s correction. “*ns”*, not significant.

In the published article, there was an error in Figure S2 as published. There was a grammatical error in the figure legend. The corrected Figure S2 and its caption appear in the Supplementary Material.

In the published article, there was an error in Figure S4 as published. There was a mistake in the figure title and the caption. The corrected Figure S4 and its caption appear in the Supplementary Material.

In the published article, there was an error in Figure S6 as published. There was a grammatical error in the caption. The corrected Figure S6 and its caption appear in the Supplementary Material.

In the published article, there was an error in Figure S8 as published. There was an extra word “classical” in the figure caption. The corrected Figure S8 and its caption appear in the Supplementary Material.

The authors apologize for these errors and state that this does not change the scientific conclusions of the article in any way. The original article has been updated.

