# Supplementary material for: Neuro-PASC is characterized by enhanced CD4+ and diminished CD8+ T cell responses to SARS-CoV-2 Nucleocapsid protein
Source: Front Immunol. 2023 May 29;14:1155770. doi: 10.3389/fimmu.2023.1155770 (PMC10258318; doi:10.3389/fimmu.2023.1155770)
Supplement: Supplementary file 1 [file DataSheet_1.docx]

**Supplementary Materials**

**
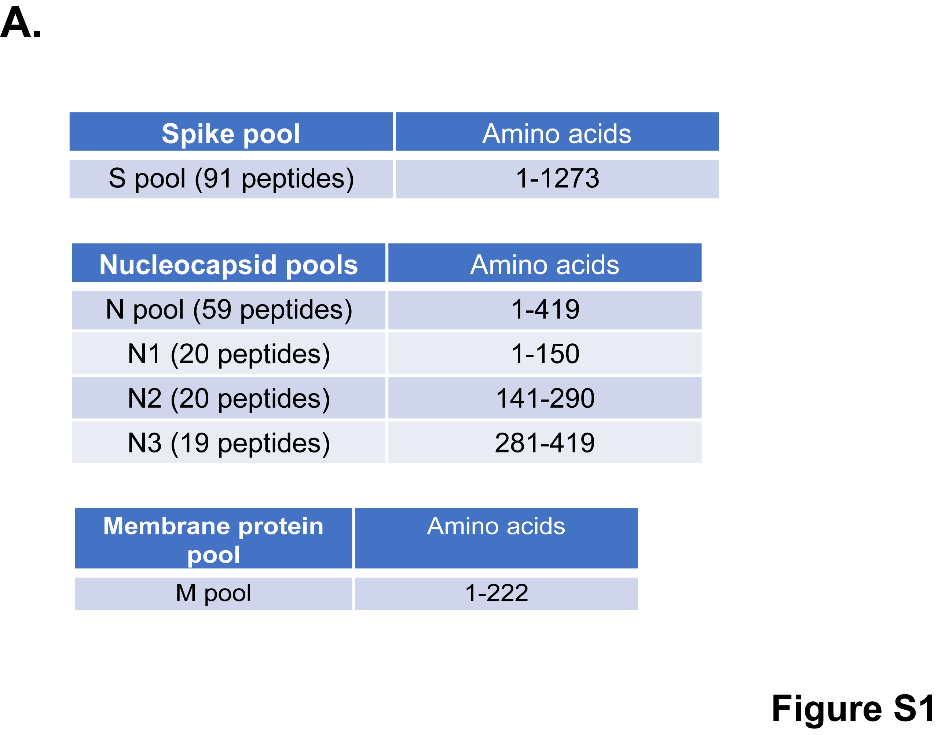
**

**Figure S1: SARS-CoV-2 peptides used in study**

Peptide pools derived from S, N, and M proteins from USA-WA1/2020 strain of SARS-CoV-2 (BEI Resources).

**
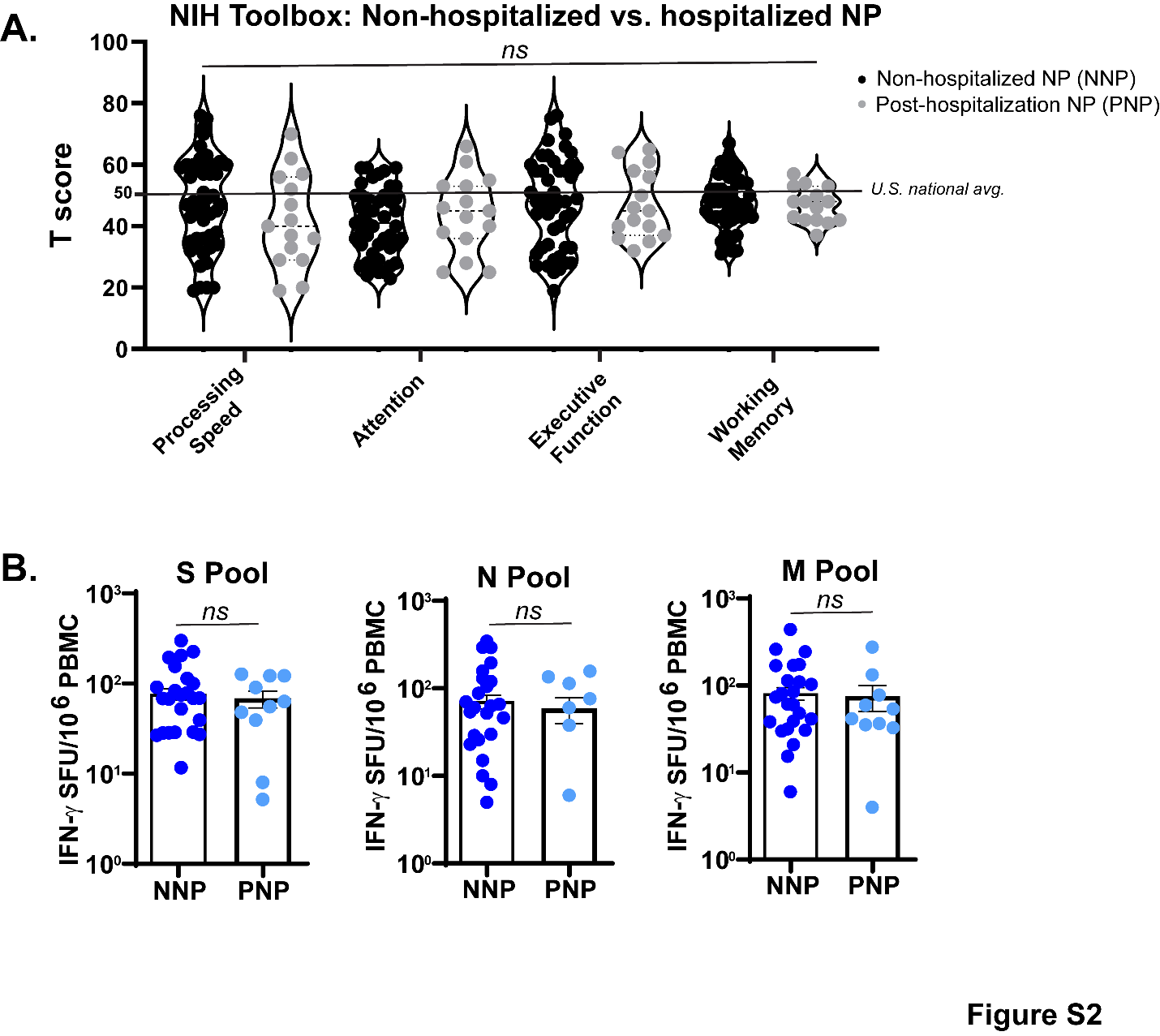
**

**Figure S2: Non-hospitalized and post-hospitalization PASC patients have similar cognitive scores and T cell responses to SARS-CoV-2 antigens.**

A.) NNP and PNP groups score similarly on Processing Speed, Attention, Executive Function, and Working Memory modules by NIH Toolbox. B.) Non-hospitalized Neuro-PASC (NNP) and post-hospitalization Neuro-PASC (PNP) patients did not exhibit differences in IFN-γ responses after stimulation with SARS-CoV-2 S, N, or M pools. Data representative of 10 experiments with all conditions plated in duplicate.

**
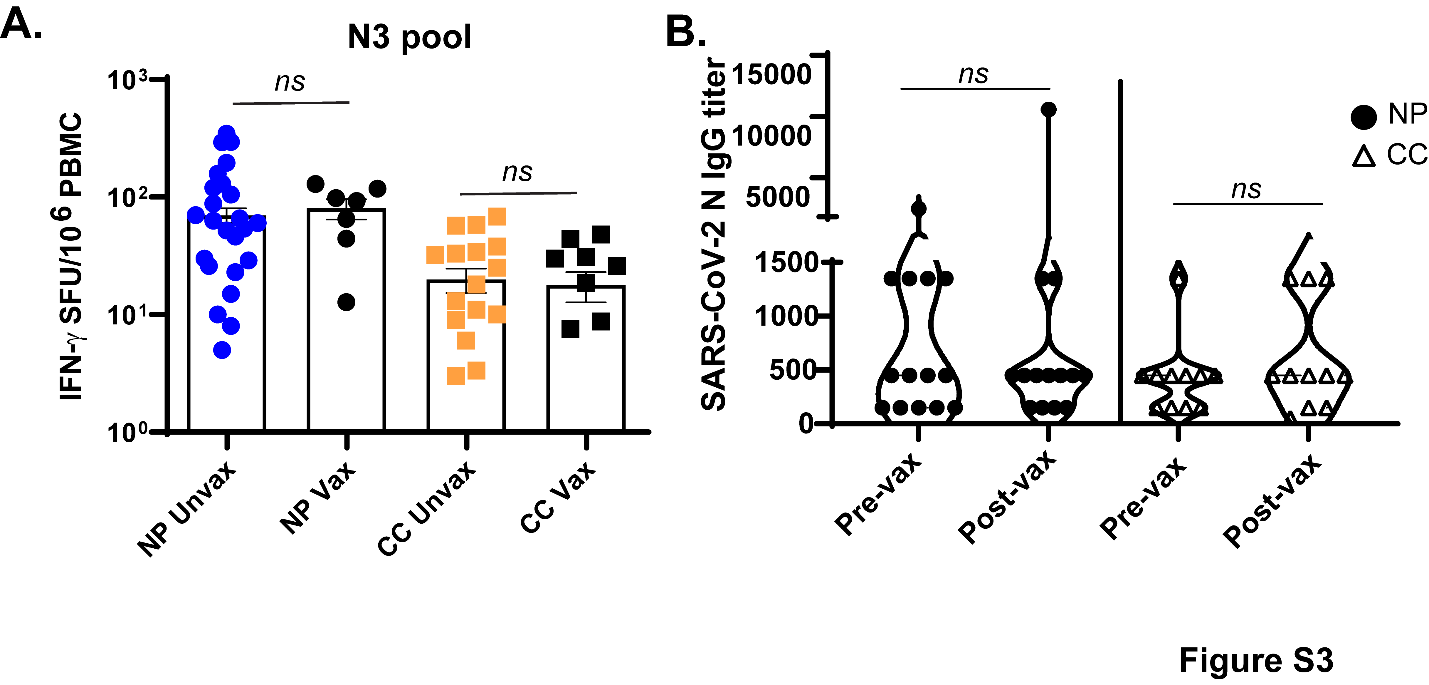
**

**Figure S3: Pfizer and Moderna SARS-CoV-2 Spike mRNA vaccines do not impact T and B cell responses to N protein in NP and CC.**

A.) Unvaccinated and vaccinated NP and CC subjects have similar IFN-γ responses to the C-terminal region of Nucleocapsid (N3). B.) Anti-N antibody responses were assessed in the same NP or CC patient pre-vaccination and at 3 months post-vaccination. No significant differences were found. Data representative of 5 independent experiments with all conditions plated in duplicate for A.

**
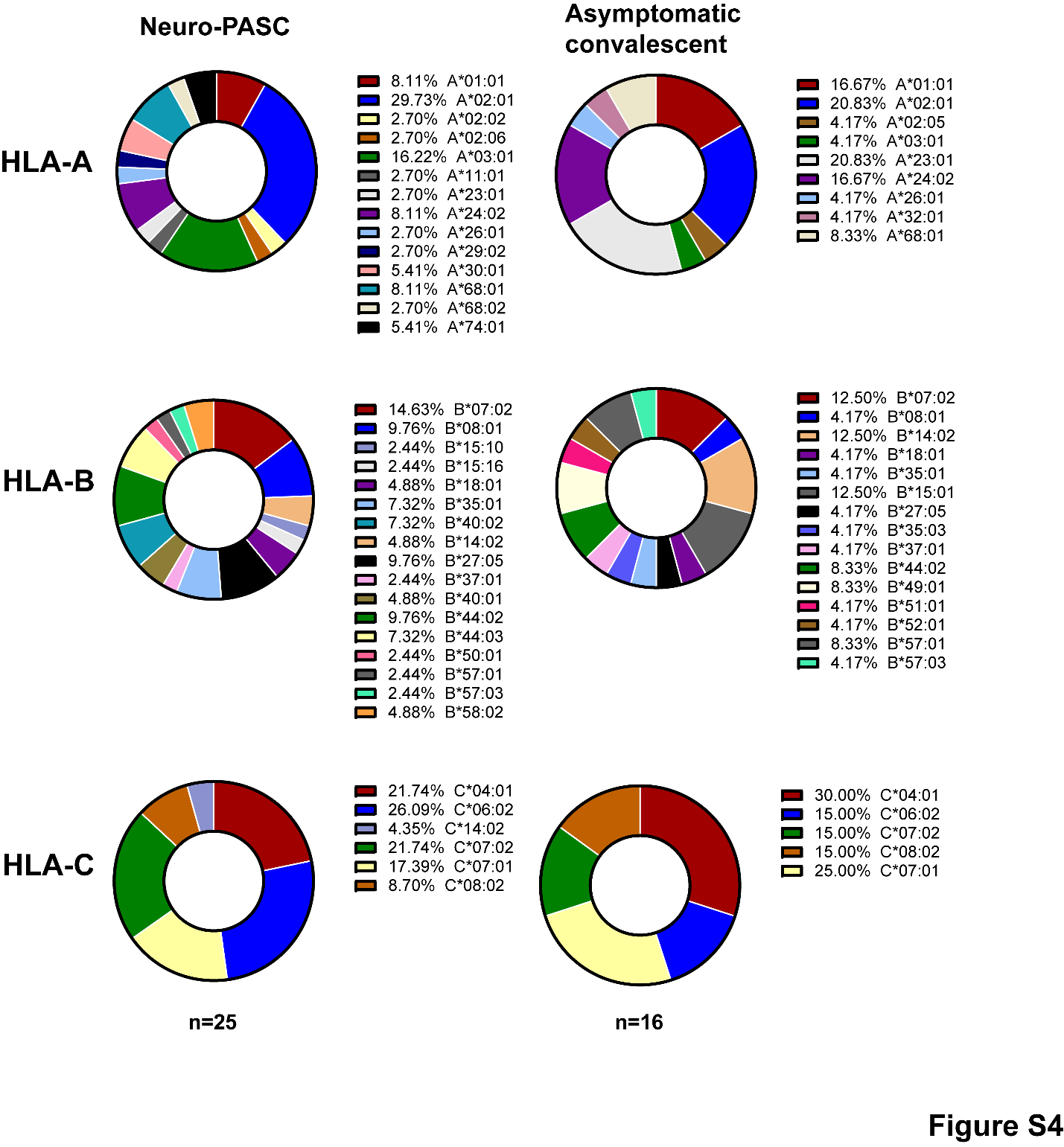
**

**Figure S4: HLA-A, -B, & -C typing in Neuro-PASC patients and convalescent controls.** NP patients have more HLA-A diversity than CC subjects. No significant differences in HLA-B*07-02 expression were found between groups, though the allele frequency trended higher in NP patients.

**
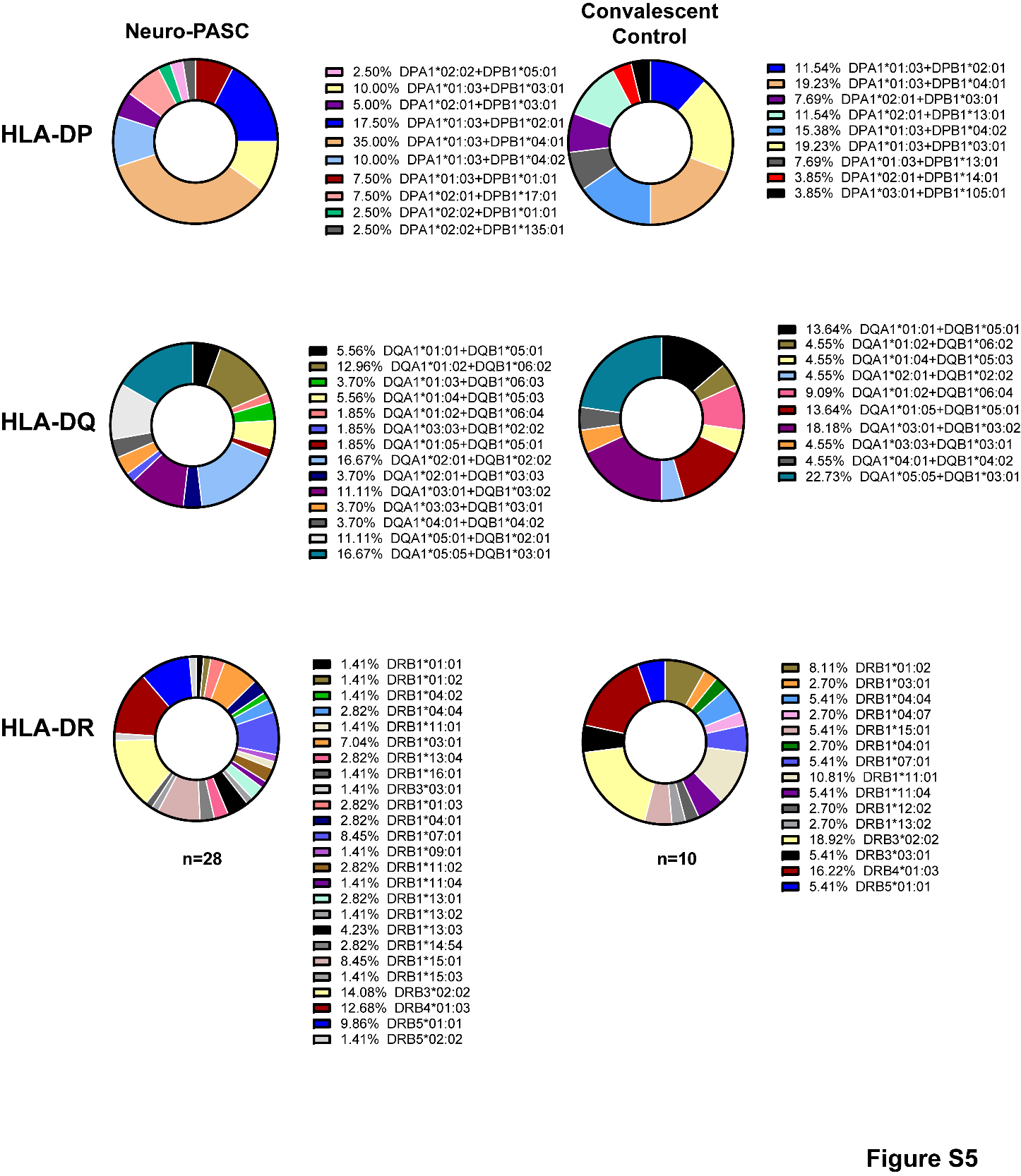
**

**Figure S5: HLA-DP, -DQ, and -DR typing in Neuro-PASC patients and convalescent controls.** No significant skewing in HLA-DP, -DQ, or -DR expression was found between NP and CC groups.


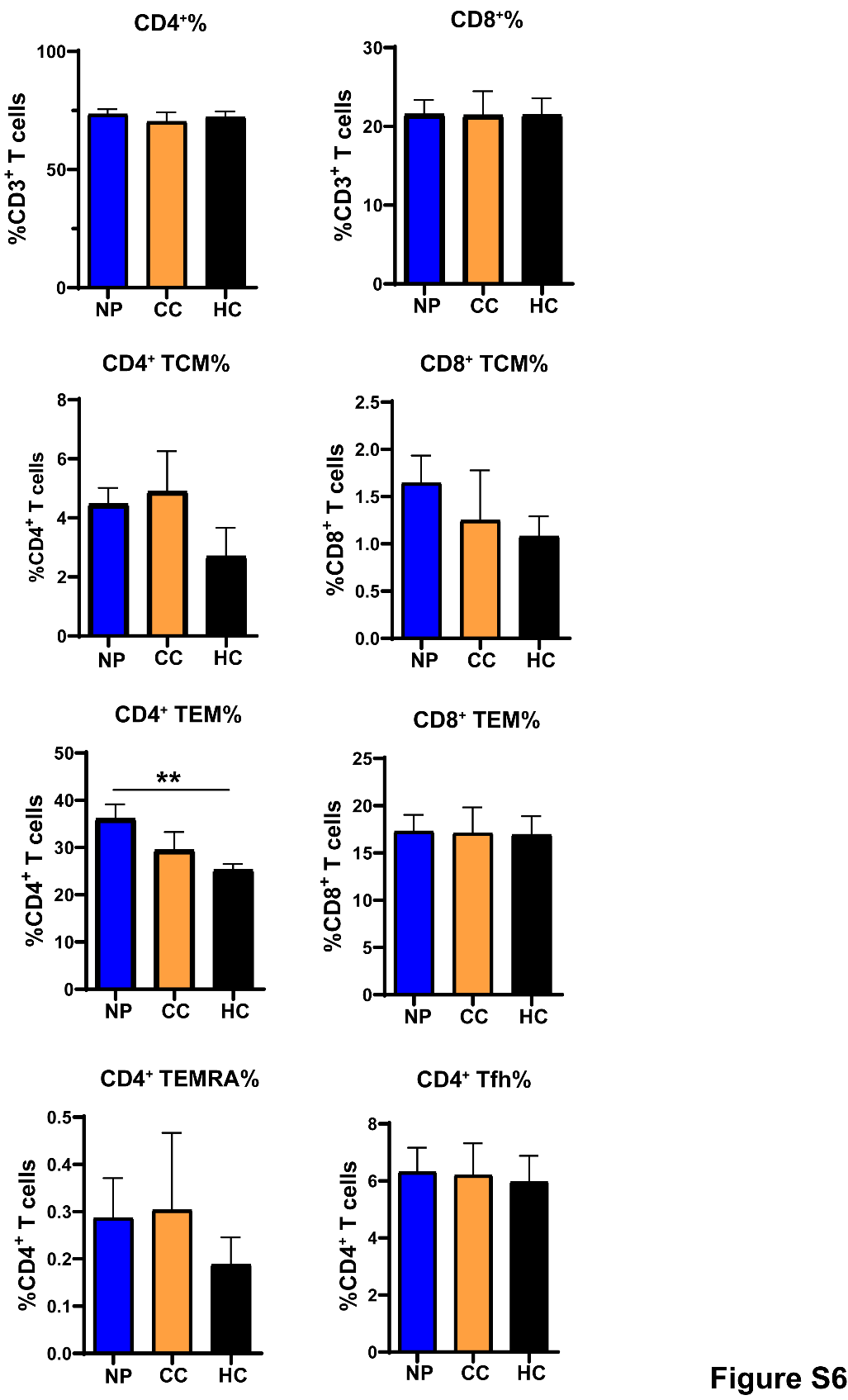


**Figure S6: Total percentages of unstimulated CD4^+^ and CD8^+^ T cell subsets between groups.**

NP, CC, and HC groups displayed no significant differences in percentages of total CD8^+^, CD8^+^ TCM or TEM cells. While there was a significant increase in CD4^+^ TEM cells as a percentage of total CD4^+^ T cells in NP vs. HC, no other cell subsets including CD4^+^ TCM, TEMRA, or Tfh cells were significantly different between groups.

**
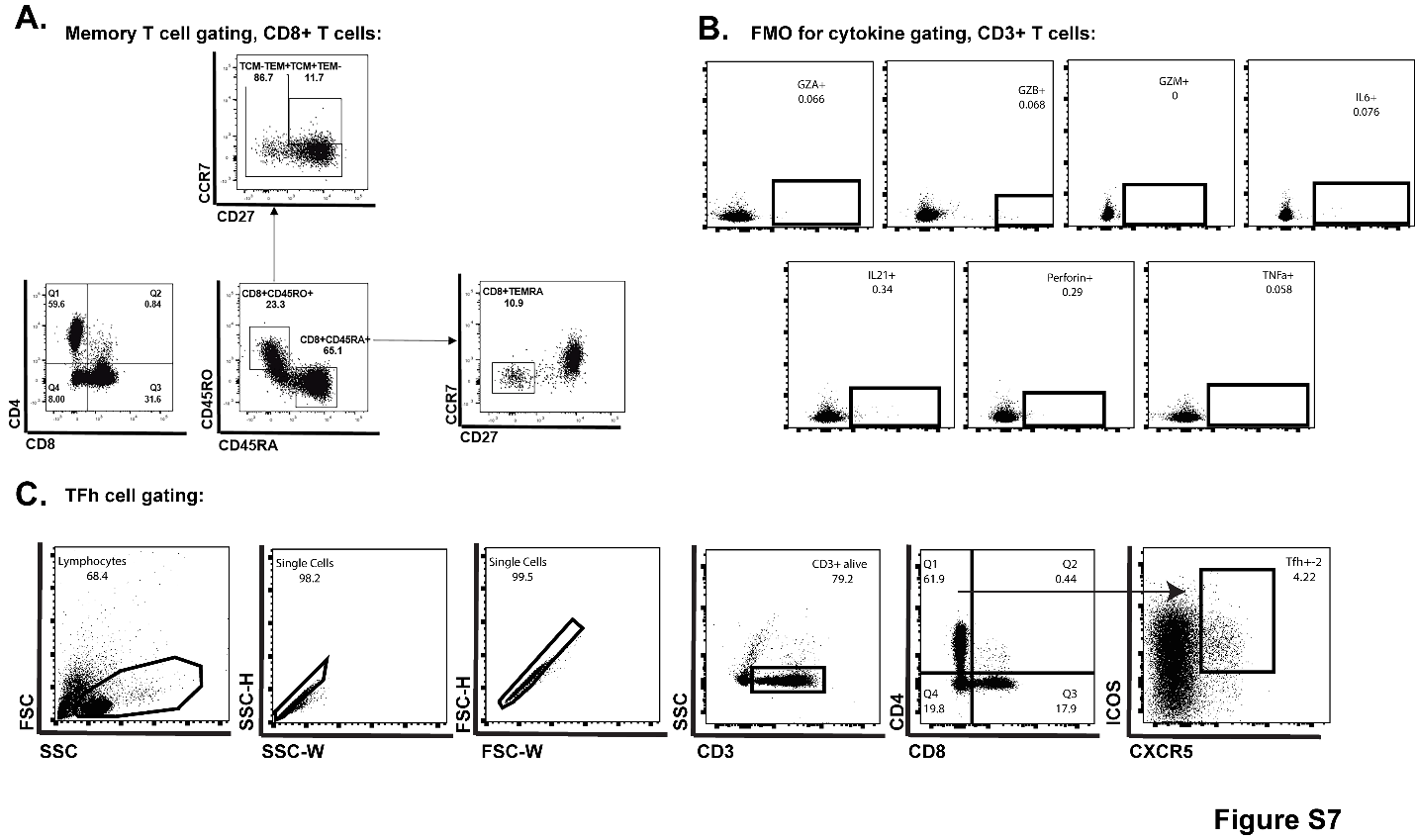
**

**Figure S7: Gating strategies and fluorescence-minus-one (FMOs) for cytokine production**

A.) Gating strategy for memory T cells by CD45RA/RO, CCR7, and CD27 expression. B.) FMOs used for gating in determining cytokine positive T cells. C.) Gating strategy for CD4^+^ Tfh cells.


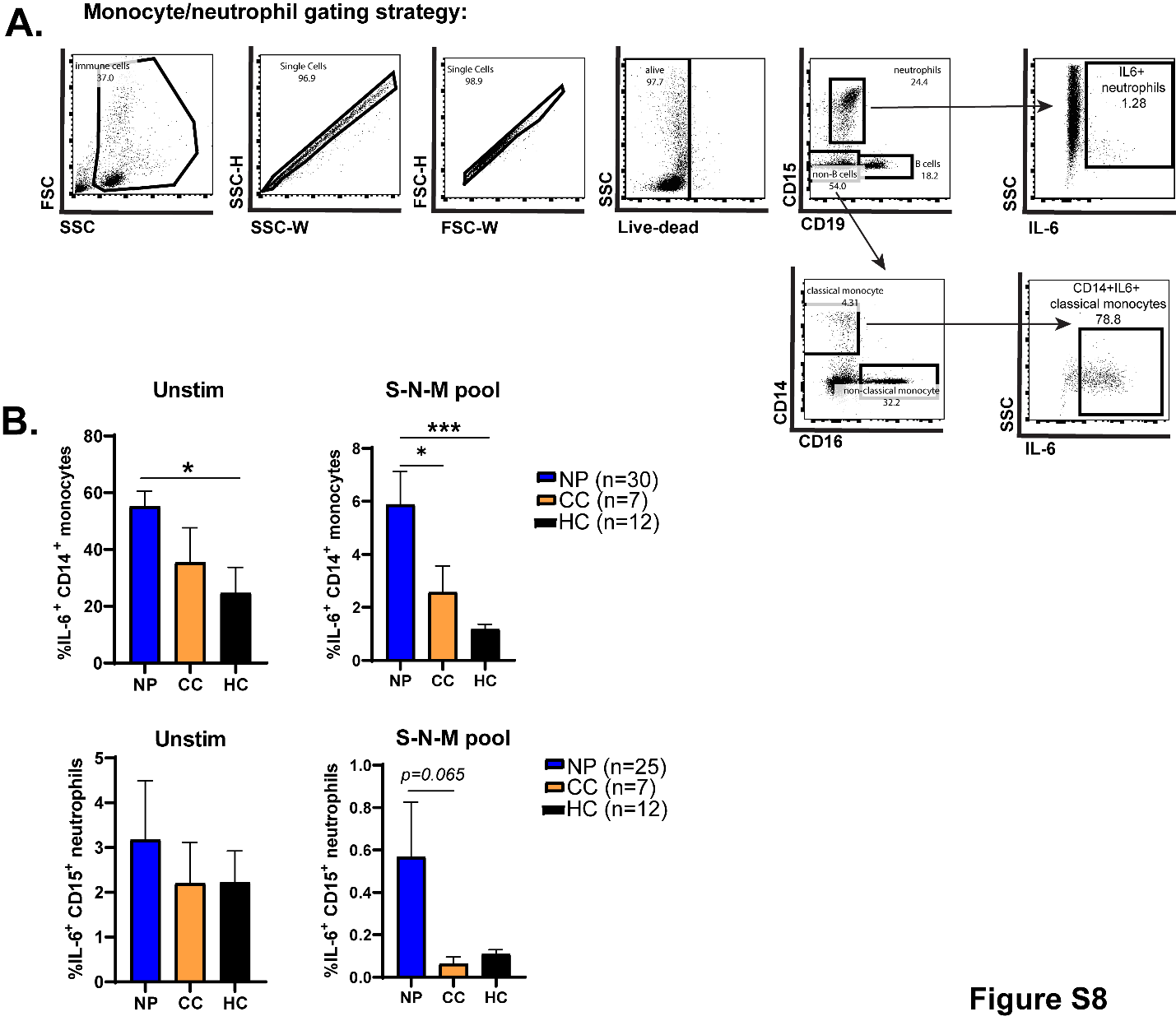


**Figure S8: Monocyte/neutrophil production of IL-6**

A.) Gating strategy for quantifying IL-6 expression in CD14^+^ classical monocytes and in CD15^+^ neutrophils. B.) Elevated IL-6 production in monocytes and neutrophils after stimulation with viral peptides in Neuro-PASC patients vs. convalescent controls.
